# Different alpha crystallin expression in human age-related and congenital cataract lens epithelium

**DOI:** 10.1186/s12886-016-0241-1

**Published:** 2016-05-28

**Authors:** Jing Yang, Sheng Zhou, Minfei Guo, Yuting Li, Jianjun Gu

**Affiliations:** State Key Laboratory of Ophthalmology, Zhongshan Ophthalmic Center, Sun Yat-sen University, 54S Xianlie, Guangzhou, Guangdong 510060 People’s Republic of China; Department of ophthalmology, The First People’s Hospital of Foshan, Guangdong Province, China; Department of Ophthalmology, Huichang County People’s Hospital, Jiangxi, China

**Keywords:** Alpha-crystallin, Age-related cataract, Congenital cataract, Lens epithelium

## Abstract

**Background:**

The purpose of this study was to investigate the different expressions of αA-crystallin and αB-crystallin in human lens epithelium of age-related and congenital cataracts.

**Methods:**

The central part of the human anterior lens capsule approximately 5 mm in diameter together with the adhering epithelial cells, were harvested and processed within 6 hours after cataract surgery from age-related and congenital cataract patients or from normal eyes of fresh cadavers. The mRNA and soluble protein levels of αA-crystallin and αB-crystallin in the human lens epithelium were detected by real-time PCR and western blots, respectively.

**Results:**

The mRNA and soluble protein expressions of αA-crystallin and αB-crystallin in the lens epithelium were both reduced in age-related and congenital cataract groups when compared with the normal control group. However, the degree of α-crystallin loss in the lens epithelium was highly correlated with different cataract types. The α-crystallin expression of the lens epithelium was greatly reduced in the congenital cataract group but only moderately decreased in the age-related cataract group. The reduction of αA-crystallin soluble protein levels in the congenital cataract group was approximately 2.4 fold decrease compared with that of the age-related cataract group, while an mRNA fold change of 1.67 decrease was observed for the age-related cataract group. Similarly, the reduction of soluble protein levels of αB-crystallin in the congenital cataract group was approximately a 1.57 fold change compared with that of the age-related cataract group. A 1.75 fold change for mRNA levels compared with that of the age-related cataract group was observed.

**Conclusions:**

The results suggest that the differential loss of α-crystallin in the human lens epithelium could be associated with the different mechanisms of cataractogenesis in age-related versus congenital cataracts, subsequently resulting in different clinical presentations.

## Background

Cataract is one of the main causes of blindness and can be congenital or acquired [1]. In developed and developing countries, the age-related cataracts occupy the leading reason of blindness [2–4]. Compared with the age-related cataracts, congenital cataracts have much less morbidity, but they are still responsible for 10 %–30 % of blindness cases in children all over the world [5]. Evidences showed that the damaged and modified lens proteins aggregate abnormally and then result in clumping in cataract lenses, which causes the light scatters and interferes the light focus [6]. Human lens proteins are mainly composed of α-, β- and γ-crystallins. Of the three types of crystallins, α-crystallin is the major type, it is composed of two subunits designated A and B, each of which has a high concentration in lens fiber cells but a low concentration in the lens epithelium [7–9]. In addition to the structural role, α-crystallin also functions as a chaperone for maintenance of lens transparency [10–12]. Alpha-crystallin in the nucleus shows age-related decreases in chaperone function. The αA-crystallin knockout studies and analysis of αA- and αB-crystallin mutative cataracts also suggest the chaperone role of these two subunits [13–16]. The lens proteins, especially in the central part of the lens, unfold and denature with aging. These unfolded or denatured proteins are prone to aggregation. Alpha-crystallin, acting as chaperone, can selectively bind to the unfolded or denatured proteins and then suppress nonspecific aggregation [11]. Experiments in the bovine, monkey and human lens provide additional evidence that alpha-crystallin prevents nonspecific aggregation in the intact lens [12, 17–19]. Differentiated lens fiber cells synthesize an abundance of α-crystallins, most studies focused on the expressions or functions of α-crystallins in lens fiber cells. However, little is known about their expressions and functions in the lens epithelium.

The lens epithelial cells cover the anterior lens and continue to differentiate into new fiber cells throughout life, however this capability declines with age [20]. Lens epithelial cells play the key role of transport and cell maintenance throughout life, in addition to this, they are the primary source of metabolic activity in the lens as well. Moreover, the lens epithelial cell integrity and survival are critical for lens transparency [7]. Although the low epithelial cell density has not been demonstrated to be one of the causes of the cataract development [21], the epithelial density decrease with aging has been documented [22]. The epithelial cells are the first defense line of lens against oxidative insults [7]. Because of the critical role of the epithelial layer integrity on normal lens physiology, apoptosis or any other abnormalities in the cytoskeleton proteins in epithelial cell induced cell death are detrimental to the underlying fiber cells and finally affect the whole lens transparency [20]. A recent review has summarized the role of αA- and αB-crystallin in the lens epithelium, they emphasized the crucial role of α-crystallin expression for the survival, growth, and proliferation of this group of cells [7].

In our study, we measured the soluble α-crystallin expression levels in the lens epithelium of age-related and congenital cataracts. The results suggest that the different clinical appearances of age-related and congenital cataracts are linked to variant αA-crystallin and αB-crystallin expressions in the lens epithelium.

## Methods

### Patients

One hundred and twenty Han Chinese patients, 60 aged 50–75 years with age-related cortical cataracts and 60 aged 1–10 years with congenital cataracts, participated in this study. The human lens epithelium specimens were collected during the cataract surgery and were used for the experiments within 6 hours. Each group was randomly divided into three subgroups (20 samples in each subgroup), with three repeats, both for western blots and real time PCR assays. Twelve normal lenses from young cadaver eyes, ages from 18 to 40 years, (collected within 6 hours from death) served as controls in the study. The samples of the control group were divided into three subgroups (4 samples in each subgroup), for three repeats, both for western blots and real time PCR assays. Ethical approval was obtained from the Institutional Review Board/Ethics Committee of Sun Yat-sen University (SYSU-ZOC-IRB) before the study was initiated. We certify that the study was performed in accordance with the Declaration of Helsinki. Informed consent was obtained from the patients or the guardians for those individuals under 16 years old. The cadaver eye tissues were obtained from the Eye Bank of Zhongshan Ophthalmic Center (Sun Yat-sen University, Guangzhou, Guangdong, China). The Institutional Review Board/Ethics Committee of Sun Yat-sen University (SYSU-ZOC-IRB) granted permission for obtaining cadaver tissues from the eye bank of Zhongshan Ophthalmic Center, and the deceased patients before their death, or their next of kin, had provided consent for these eye tissues to be used in research.

### Lens epithelium specimen collection

The human lens anterior capsule specimens with the adhering epithelium approximately 5 mm in diameter, were obtained from cataract surgery and placed in Eppendorf® tubes for further use.

### Lens epithelium sample RNA extraction and real-time PCR:

TRIzol® (Invitrogen™ Life Sciences, Thermo Fisher, Wilmington, DE, USA) was used to isolate the total RNA from the human lens epithelium specimens according to the manufacturer’s instructions. Quality assessment and concentration of RNA extracts was done by NanoDrop® Products (Thermo Fisher) before cDNA preparation. The Superscript First-strand Synthesis Kit (Invitrogen™, Thermo Fisher) was used for the cDNA synthesis. Reactions with the same volume of cDNA were prepared using SYBR® Green Master Mix (Bio-Rad, Hercules, CA, USA) and the Bio-Rad CFX96 real time system was used for the quantitative real-time PCR analyses. We repeated each reaction in triplicate and at least three times for each experiment to confirm reproducibility. The threshold cycle (Ct) values for each gene were analyzed using the standard curve method. The CT values were normalized to the expression of GAPDH and the log of the average relative expression ± SEM was reported. The gene specific primer sequences are listed in Table 1.

**Table 1:** Sequences of related primers for real-time PCR

Primer	Forward	Reverse
αA-crystallin	5'-GAGATCCACGGAAAGCACAAC-3'	5'-GGTAGCGGCGGTGGAACT-3'
αB-crystallin	5'-CTTTGACCAGTTCTTCGGAG-3'	5'-CCTCAATCACATCTCCCAAC-3'
GAPDH	5'-CCACATCGCTCAGACACCAT-3'	5'-GGCAACAATATCCACTTTACCAGAGT-3'

### Lens epithelium protein extraction and western blots

We used a radioimmune precipitation assay buffer with a protease inhibitor mixture, phenylmethanesulfonyl fluoride (PMSF), and sodium orthovanadate (Santa Cruz Biotechnology, Santa Cruz, CA, USA) for the lysis of the human lens epithelium specimens. The lysis buffer together with the specimens were sonicated and then centrifuged at 13,000 × g for 10 min. The supernatant was collected as the protein samples and the determinations of the protein concentrations were performed by the Bradford procedure (Bio-Rad) and by western blotting. The proteins were separated on 12 % sodium dodecyl sulfate polyacrylamide gels at 110 V for 60 min and then transferred onto nitrocellulose membranes at 100 V for 150 min. After being blocked in 5 % powdered milk solution for 60 min at room temperature, the membranes were incubated with appropriate primary antibodies overnight at 4 °C and then with secondary antibody for 60 min at room temperature. Beta-actin was used as the internal control. The anti-αA-crystallin antibody (sc-22389; Santa Cruz Biotechnology) was used at a 1:200 dilution. The anti-αB-crystallin antibody (sc-22744; Santa Cruz Biotechnology) was used at a 1:200 dilution. The peroxidase-based detection was performed with Chemiluminescence Reagent (NEN Life Science, Xinhailing Company, Shenzhen, China). Each experiment was repeated three times. We performed densitometric analyses of the western blots. The optical density (O.D.) values of the various bands were analyzed using a computer that was equipped with image analysis software (Image J; National Institutes of Health, https://imagej.nih.gov/ij/).

### Statistical analyses

Statistical analyses were performed according to the sample size and sample group as previously described in the methods. All values were expressed as means ± standard deviation (SD). One way ANOVA (Prism 3 software; GraphPad, La Jolla, CA, USA) was performed for the three groups. P values of < 0.05 were considered as statistically significant results.

## Results

### Decreased αA-crystallin and αB-crystallin mRNA levels in the lens epithelium of age-related and congenital cataracts

**Fig. 1:**
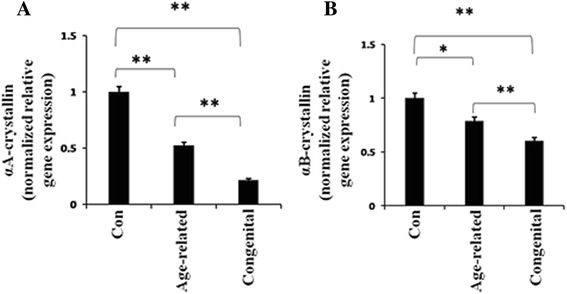
αA- and αB-crystallin relative gene expressions in age-related and congenital cataract lens epithelium. RNA were extracted from human lens capsule epithelium specimens. Real-time PCR was performed for detecting the RNA levels of αA-(**a**) and αB-crystallin (**b**) in each group. GAPDH was used as the internal control gene. (mean ± SD, *n* = 3). *P < 0.05 **P < 0.001

To investigate the gene expression of αA-crystallin and αB-crystallin in the lens epithelium of age-related and congenital cataracts, we detected the mRNA expression levels by real-time PCR assays. Total RNA was extracted from the human lens epithelium specimens. Both αA-crystallin and αB-crystallin expression levels were significantly reduced in the age-related and congenital cataract groups compared with the normal control group. However for αA-crystallin in the age-related cataract group, the gene expression was approximately 0.55 fold that of the normal control, and αA-crystallin in the congenital cataract group was 0.25 fold that of the normal control group (Fig. 1a). The reduction of αA-crystallin gene expression in the congenital cataract group was approximately 1.67 times that of the age-related cataract group (Fig. 1a). For αB-crystallin in the age-related cataract group, the gene expression level was approximately 0.8 fold that of the normal control, but αB-crystallin gene expression levels in the congenital cataract group were only 0.65 fold that of the control group (Fig. 1b). The reduction of αB-crystallin gene expression was about 1.75 times greater in the congenital cataract group than in the age-related cataract group (Fig. 1b). These results indicated that the reduction of both αA-crystallin and αB-crystallin transcripts differed dramatically in the lens epithelium of the age-related versus congenital cataracts. The two gene expression levels were more significantly reduced in the congenital cataract group.

### Decreased soluble αA-crystallin and αB-crystallin protein levels in the lens epithelium of age-related and congenital cataracts

**Fig. 2:**
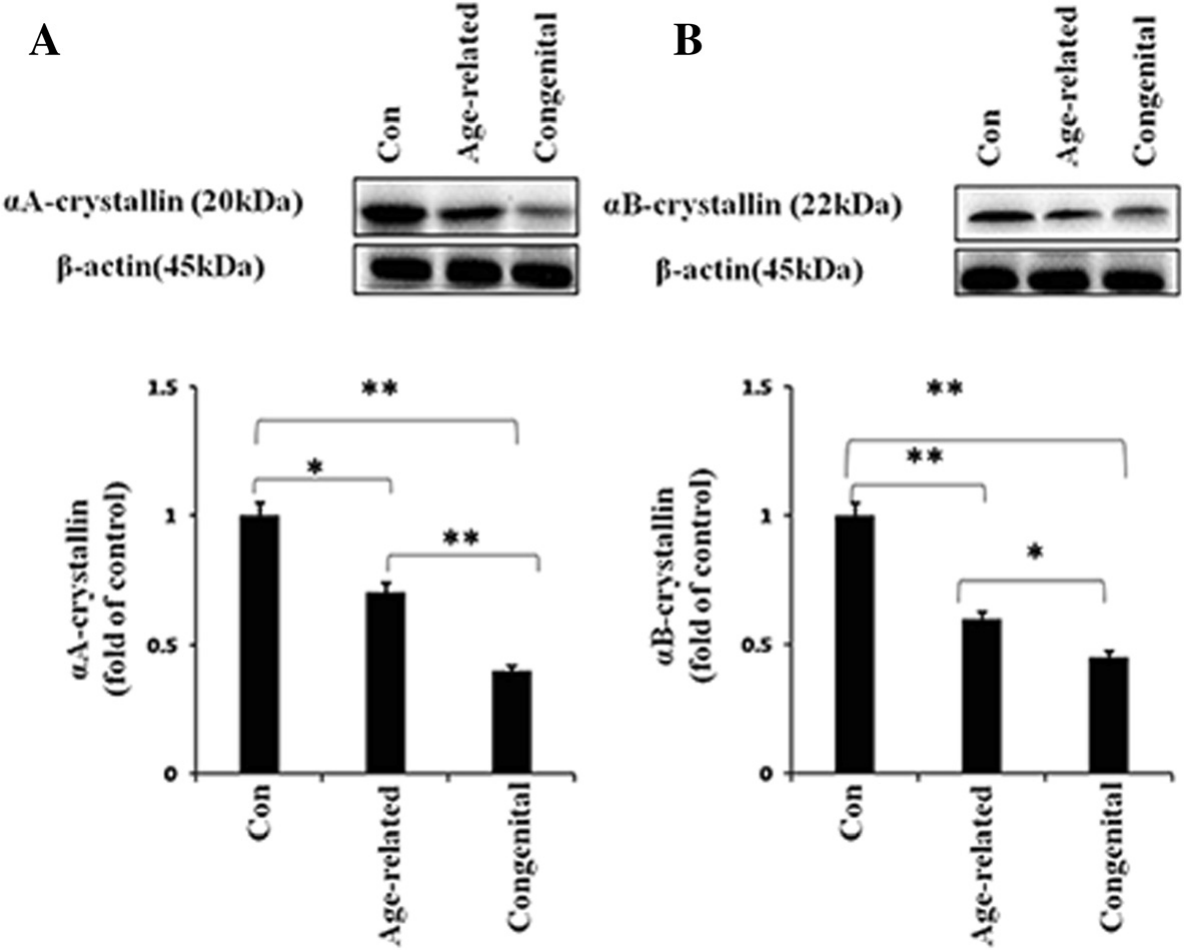
Soluble αA- and αB-crystallin protein expressions in age-related and congenital cataract lens epithelium. Proteins were extracted from human lens capsule epithelium specimens. Western blot assay was performed for detecting the αA- (**a**) and αB-crystallin (**b**) protein levels of each group. β-actin was used as the internal control. (mean ± SD, *n* = 3). *P < 0.05 **P < 0.001

Based on the gene expression changes of αA-crystallin and αB-crystallin in age-related and congenital cataract lens epithelium, we then determined if a similar trend could be found for the protein levels. The soluble protein levels of αA-crystallin and αB-crystallin were assessed by western blots. The results showed the protein levels of both soluble αA-crystallin and αB-crystallin were significantly reduced in age-related and congenital cataracts when compared with the normal control group. Soluble αA-crystallin in the age-related cataract was approximately 0.75 fold that of the normal control group, and in the congenital cataract it was approximately 0.4 fold that of the normal control group (Fig. 2a). In age-related cataracts, the protein levels of soluble αB-crystallin were approximately 0.65 fold that of the normal control group, and in the congenital cataract group, the levels were approximately 0.45 fold that of the normal control group (Fig. 2b). In addition to the significant differences noted between the cataract and normal control groups, differences were also noted in comparisons between the two different types of cataracts. The protein reduction level of soluble αA-crystallin in the congenital cataract group was approximately 2.4 fold that of the age-related cataract group. The protein reduction levels of soluble αB-crystallin in the congenital cataract group was approximately 1.57 fold that observed in the age-related cataract group. These results showed similar alteration patterns of soluble α-crystallin protein and mRNA levels.

## Discussion

We present here the data on the levels of αA-crystallin and αB-crystallin protein and mRNA in the lens epithelium of human age-related and congenital cataracts, compared with control lenses. The lens arises from invagination of the head ectoderm during embryonic development, comprising two cell types, the epithelial and fiber cells [23]. The lens epithelium is the only tissue in the body that grows throughout life. Lens epithelial cells are responsible for the growth and development of the entire ocular lens. In our study, the reductions of αA-crystallin and αB-crystallin mRNA and soluble protein levels were identified in the lens epithelium of age-related and congenital cataracts compared with the normal control lens. The data may help to provide an explanation for the cause of cataractogenesis. The reduced levels of αA-crystallin and αB-crystallin, especially the soluble crystallin forms, could disturb the normal homeostasis of the lens epithelium, and this could have a role in the survival and proliferation of these cells [7]. The first line of defense against oxidative insults could then become compromised and even the underlying fiber cells would be affected [24], thus contributing to cataract formation.

As described above, our data indicate that changes of the expression levels of αA-crystallin and αB-crystallin in the lens epithelium might be involved in cataractogenesis. Many other issues could also be involved in cataractogenesis, such as during the developmental timing process. Additional underlying risk factors and clinical appearances are also important in cataractogenesis. Because the cataractogenesis process is quite different between age-related and congenital cataracts, the regulatory factors may differ accordingly. More interestingly, we also found that the reduction of the two soluble crystallin proteins in the lens epithelium was significantly greater in the congenital cataract than in the age-related cataract group. The results indicate that the differences in reduction of α-crystallin might be one of the contributing factors to the variety of cataract presentation. Because of the highly reduced expression of α-crystallin in the lens epithelium of congenital cataracts, the fiber cells differentiated from these lens epithelial cells may lack α-crystallin. Thus, the dramatic changes in cell shape and length of these differentiated fiber cells, which closely rely on an intact cytoskeleton, would be adversely affected [24]. In addition, α-crystallin is required for the stabilization of the microtubules, actin, and intermediate filament cytoskeleton structures [25]. All these changes could promote cataract formation.

In conclusion, we demonstrated that the expressions of soluble αA-crystallin and αB-crystallin protein and mRNA in the lens epithelium were reduced both in the age-related and the congenital cataract patients. Furthermore, the reduction was more severe in the congenital cataract. These findings indicate the reduction level of soluble α-crystallin in the lens epithelium might be one of the contributing factors that lead to the different appearances of age-related versus congenital cataract. However, the genotypes of the patients from which samples were obtained were not included in these studies, therefore, crystallin expression changes might not be the primary cause of cataractogenesis. Changes in α-crystallin or in another gene, including mutations, could be contributing factors for cataract formation. In the future, α-crystallin mutation studies in different types of cataracts should be done, and further studies defining the specific interactions of the cell matrix and signaling proteins with α-crystallin may reveal the mechanism by which α-crystallin regulates different aspects of lens epithelial cell survival, proliferation, and differentiation. Therefore, a more detailed study of the underlying relationships between cataract formation and α-crystallin expression is needed.

## Conclusions

The results suggest that the differential loss of α-crystallins in the human lens epithelium might be associated with the different cataractogenesis patterns found in the age-related and congenital cataract groups, which subsequently results in different clinical presentations.

## Abbreviations

GAPHD, glyceraldehyde 3-phosphate dehydrogenase.

## References

[1] Li FF (2008). Nonsense mutation in the CRYBB2 gene causing autosomal dominant progressive polymorphic congenital coronary cataracts. Mol Vis.

[2] Prokofyeva E, Wegener A, Zrenner E (2013). Cataract prevalence and prevention in Europe: a literature review. Acta Ophthalmol.

[3] Huang W (2012). Five-year incidence and postoperative visual outcome of cataract surgery in urban southern China: the Liwan Eye Study. Invest Ophthalmol Vis Sci.

[4] Richter GM (2012). Risk factors for incident cortical, nuclear, posterior subcapsular, and mixed lens opacities: the Los Angeles Latino eye study. Ophthalmology.

[5] Ding X (2014). A novel MIP gene mutation analysis in a Chinese family affected with congenital progressive punctate cataract. PLoS One.

[6] Sakaue H (2014). Alpha B- and betaA3-crystallins containing d-Aspartic acids exist in a monomeric state. Biochim Biophys Acta.

[7] Andley UP (2008). The lens epithelium: focus on the expression and function of the alpha-crystallin chaperones. Int J Biochem Cell Biol.

[8] Andley UP (2007). Crystallins in the eye: Function and pathology. Prog Retin Eye Res.

[9] Wang X (2004). Expression and regulation of alpha-, beta-, and gamma-crystallins in mammalian lens epithelial cells. Invest Ophthalmol Vis Sci.

[10] Bloemendal H (2004). Ageing and vision: structure, stability and function of lens crystallins. Prog Biophys Mol Biol.

[11] Horwitz J (2003). Alpha-crystallin. Exp Eye Res.

[12] Rao PV (1995). Evidence that alpha-crystallin prevents non-specific protein aggregation in the intact eye lens. Biochim Biophys Acta.

[13] Hayes VH, Devlin G, Quinlan RA (2008). Truncation of alphaB-crystallin by the myopathy-causing Q151X mutation significantly destabilizes the protein leading to aggregate formation in transfected cells. J Biol Chem.

[14] Xi JH (2008). Mechanism of small heat shock protein function in vivo: a knock-in mouse model demonstrates that the R49C mutation in alpha A-crystallin enhances protein insolubility and cell death. J Biol Chem.

[15] Brady JP (2001). AlphaB-crystallin in lens development and muscle integrity: a gene knockout approach. Invest Ophthalmol Vis Sci.

[16] Andley UP, Hamilton PD, Ravi N (2008). Mechanism of insolubilization by a single-point mutation in alphaA-crystallin linked with hereditary human cataracts. Biochemistry.

[17] Wang K, Spector A (1994). The chaperone activity of bovine alpha crystallin. Interaction with other lens crystallins in native and denatured states. J Biol Chem.

[18] Boyle D, Takemoto L (1994). Characterization of the alpha-gamma and alpha-beta complex: evidence for an in vivo functional role of alpha-crystallin as a molecular chaperone. Exp Eye Res.

[19] Carver JA (1996). Age-related changes in bovine alpha-crystallin and high-molecular-weight protein. Exp Eye Res.

[20] Sharma KK, Santhoshkumar P (2009). Lens aging: effects of crystallins. Biochim Biophys Acta.

[21] Harocopos GJ (1998). Human age-related cataract and lens epithelial cell death. Invest Ophthalmol Vis Sci.

[22] Balaram M (2000). Noncontact specular microscopy of human lens epithelium. Invest Ophthalmol Vis Sci.

[23] Martinez G, de Iongh RU (2010). The lens epithelium in ocular health and disease. Int J Biochem Cell Biol.

[24] Bassnett S (2002). Lens organelle degradation. Exp Eye Res..

[25] Quinlan R (2002). Cytoskeletal competence requires protein chaperones. Prog Mol Subcell Biol.

